# Essential oil composition of *Eucalyptus microtheca* and *Eucalyptus viminalis*

**Published:** 2015

**Authors:** Malek Taher Maghsoodlou, Nasrin Kazemipoor, Jafar Valizadeh, Mohsen Falak Nezhad Seifi, Nahid Rahneshan

**Affiliations:** 1 *Department of Chemistry, University of Sistan and Baluchestan, Zahedan, Iran*; 2 *Department of Agriculture, Shiraz University, Shiraz, Iran*; 3 *Department of Biology, University of Sistan and Baluchestan, Zahedan, Iran*

**Keywords:** *Essential oil*, *Eucalyptus microtheca*, *Eucalyptus viminalis*, *Myrtaceae*, *Hydro-distillation*, *GC/MS*

## Abstract

**Objective:**

*Eucalyptus *(Fam. Myrtaceae) is a medicinal plant and various *Eucalyptus* species possess potent pharmacological actions against diabetes, hepatotoxicity, and inflammation. This study aims to investigate essential oil composition from leaves and flowers of *E. microtheca* and *E. viminalis* leaves growing in the Southeast of Iran.

**Materials and Methods:**

The aerial parts of these plants were collected from Zahedan, Sistan and Baluchestan province, Iran in 2013. After drying the plant materials in the shade, the chemical composition of the essential oils was obtained by hydro-distillation method using a Clevenger-type apparatus and analyzed by GC/MS.

**Results:**

In the essential oil of* E. microtheca* leaves, 101 compounds representing 100%, were identified. Among them, α-phellandrene (16.487%), aromadendrene (12.773%), α-pinene (6.752%), globulol (5.997%), ledene (5.665%), P-cymen (5.251%), and β-pinene (5.006%) were the major constituents. In the oil of* E. microtheca* flowers, 88 compounds representing 100%, were identified in which α-pinene (16.246%), O-cymen (13.522%), β-pinene (11.082%), aromadendrene (7.444%), α-phellandrene (7.006%), globulol (5.419%), and 9-octadecenamide (5.414%) were the major components. Sixty six compounds representing 100% were identified in the oil of *E. viminalis* leaves. The major compounds were 1, 8-cineole (57.757%), α-pinene (13.379%), limonene (5.443%), and globulol (3.054%).

**Conclusion:**

The results showed the essential oils from the aerial parts of *Eucalyptus *species are a cheap source for the commercial isolation of α-phellandrene, α-pinene, and 1, 8-cineole compounds to be used in medicinal and food products. Furthermore, these plants could be an alternative source of insecticide agents**.**

## Introduction

Plants and their derivatives such as essential oils have long been used as food flavoring, beverages, and antimicrobial agents (Ghasemi et al., 2005 ▶). Nowadays, developing countries pay more attention to herbal medicines due to the noxious side effects of synthetic medicines on patients. In addition, the application of natural antioxidants in food factories has attracted a growing interest (Asghari and Mazaheritehrani, 2010 ▶) to minimize such oxidative damages in human body. Therefore, research works concerning essential oils as potential antioxidants for treatment of human diseases and free radical-related disorders are important. Concomitantly, public attention to natural antioxidants has been increased during the last years, and it is necessary to find natural sources of antioxidants that could replace synthetic antioxidants or at least reduce their use as food additives. For these reasons, numerous researches have been conducted in the extraction field of biologically active compounds from the herbs (Shahidi, 2000 ▶). *Eucalyptus *(Fam. Myrtaceae) is a genus of evergreen aromatic flowering trees, which has over 600 species (Jahan et al., 2011 ▶; Nagpal et al., 2010 ▶). It is indigenous in Australia and its Northern islands (Mozaffarian, 1996 ▶). Because of their economic value, various species of *Eucalyptus *are cultivated in sub-tropical and warm temperate regions (Sastri, 2002 ▶). Some of the *Eucalyptus *species are used for feverish conditions (malaria, typhoid, and cholera) and skin problems such as burns, ulcers, and wounds (Reynolds and Prasad, 1982 ▶). *Eucalyptus *species contain volatile oils that are most plentiful in the plant leaves (Pearson, 1993 ▶). Anticancer, antifungal, anti-inflammatory (Sadlon and Lamson, 2010 ▶), and antioxidant properties (Grassmann et al., 2000 ▶) have been attributed to the leaf extracts of this plant. 

For this reason, the importance of these plants as an herbal medicine, the aim of the present study was to investigate the chemical composition of the essential oil from leaves and flowers of *Eucalyptus microtheca* and *E. viminalis* leaves from Zahedan (with latitude of 29° 29ˊ N and longitude of 60° 51ˊ E and 1352 m above sea level in summer of 2013) in Sistan and Baluchestan province, Iran as an important geographical zone for medicinal plants. 

## Material and Methods

**Plant materials**

*Eucalyptus microtheca* and *E. viminalis* were collected in June, 2013 from Zahedan in Sistan and Baluchestan province (GPS coordinates: 60.8628, 29.4964), Iran during the flowering stage. The taxonomic identification of each plant was confirmed by Professor V. Mozaffarian, Research Institute of Forests and Ragelands, Tehran, Iran. The voucher specimens were deposited in the national herbarium of Iran (TARI). Collected plant materials were separated with a meticulous care and dried in the shade to avoid extra damaging and minimizing cross-contamination of the plant leaves.

**Isolation of the essential oil**

The leaves and flowers of *E. microtheca* and *E. viminalis* leaves were dried and milled into a fine powder. The volatile oils were isolated by hydro-distillation method using a Clevenger-type apparatus. For the extraction, 50 g of the cleaned, air-dried and powdered of leave samples of *E. microtheca* and *E. viminalis* were hydro-distilled with 500 mL water in a Clevenger-type apparatus for 4 h. Moreover, 30 g of the *E. microtheca* flower samples were hydro-distilled with 300 mL water for 4 h. The oils were dried over anhydrous Na_2_SO_4_ (Merck), stored in a dark glass bottle and kept at -8 °C until analysis.

**Essential oil analysis**

 The essential oils were analyzed on an Agilent 6890 gas chromatograph interfaced to an Agilent 5973 N mass selective detector (Agilent Technologies, Palo Alto, USA). A fused silica capillary column (30 m length × 0.025 mm internal diameter × 0.25 μm film thickness; HP-1; silica capillary column, Agilent Technologies) was used. The data were acquired under the following conditions: The oven temperature increased from 40 °C to 250 °C at a rate of 3 °C/min.

 The temperatures of injector and detector also were 250 °C and 230 °C, respectively. The carrier gas was helium (99.999%) with a flow rate of 1 ml/min and the split ratio was 50 ml/min. For GC–MS detection, an electron ionization system with ionization energy of 70 eV was used. The retention indices were calculated for all volatile constituents using retention time of *n*-alkanes (C_8_-C_22_) which were injected at the same chromatographic conditions. The components were identified by comparing retention indices with those of standards. The results were also confirmed by comparing their mass spectra with the published mass spectra or Wiley library.

## Results

The oils were isolated by hydro-distillation and analyzed by capillary gas chromatography, using flame ionization and mass spectrometric detection. The obtained results of the identified compounds in the essential oil of leaves and flowers of *E. microtheca* and *E. viminalis* leaves with their percentage, retention index (RI), and retention time (t_R_) are shown in Tables 1, 2, and 3, respectively. The chromatographic analysis of extracted volatile oil of *E. microtheca* leaves revealed the presence of sesquiterpenes (47.852%), monoterpenes (46.844%), polyketides and fatty acids (3.496%), diterpene (0.140%), alkanes (0.085%), aromatic compounds (0.029%), and other compounds (1.521%).

**Table 1:** Composition of the volatile oil of *Eucalyptus microtheca *leaves

**No.**		**Compound**	**%** 1	**RI** 2	**RT** 3 ** (min)**
1		α –thujene	0.742	742	9.381
2		α -pinene	6.752	767	9.716
3		comphene	0.079	792	10.063
4		β - pinene	5.006	817	11.33
5		β -myrcene	0.533	850	12.025
6		α -phellandrene	16.487	871	12.755
7		α -terpinene	0.832	892	13.103
8		p- cymene	5.251	913	13.374
9		β -phellandrene	2.194	934	13.626
10		Limonene	1.503	955	13.722
11		*Cis*-ocimene	1.655	976	14.144
12		β –ocimene Y	0.101	997	14.546
13		γ -terpinene	1.235	1018	14.976
14		Cymene	0.024	1038	16.021
15		α -terpinolene	0.425	1054	16.267
16		Rosefuran	0.024	1073	16.499
17		Cycloheptanmethanol	0.061	1092	16.581
18		Linalool L	0.093	1112	16.806
19		Isoamyl isovalerate	0.529	1131	17.038
20		Isoamyl valerate	0.056	1151	17.152
21		Fenchol	0.076	1170	17.222
22		*Trans*-pinene hydrate	0.062	1190	17.598
23		Allocimene	0.049	1209	18.247
24		1-terpineol	0.045	1229	18.412
25		1-methylnorcarane	0.051	1267	19.229
26		Ethylbenzoate	0.124	1287	19.367
27		4-terpineol	1.256	1326	20.172
28		1-(adamantly) cyclohexene	0.042	1345	20.311
29		β -fenchol	0.203	1384	20.695
30		*cis*-sabinol	0.224	1404	21.183
31		Thiophene, 2-ethyl-5-methyl	0.120	1428	21.729
32		Ascaridole	0.085	1448	21.866
33		Dicyclobutylidene oxide	0.084	1527	24.404
34		Divinyldimethylsilane	0.114	1507	23.545
35		Piperitone	0.196	1487	22.992
36		1-methoxyhept-1-yne	1.809	1467	22.838
37		Citronellyl formate	0.029	1546	24.67
38		Carvacrol	0.420	1625	26.426
39		α** –**cubebene	0.160	1927	28.309
40		Isoledene	0.278	1957	29.297
41		Copaene	0.308	1987	29.387
42		2-pentene-1-ol, 2-methyl	0.215	1713	30.103
43		α –gurjunene	1.897	1762	30.826
44		*Trans*-caryophyllene	0.539	1779	31.059
45		Aromadendrene	12.773	1811	32.17
46		Epizonaren	0.067	1828	32.30
47		α –humulene	0.142	1844	32.435
48		Alloarmadendrene	2.520	1861	32.798
49		γ –gurjunene	0.327	1877	33.178
50		α –copaene	0.755	1893	33.39
51		β –selinene	0.525	1910	33.692
52		β –panasinsene	0.702	1926	33.862
53		Ledene	5.665	1943	34.303
54		α –muurolene	0.398	1959	34.357
55		Geremacrene B	0.099	1975	34.563
56		α –amorphene	1.666	1992	34.862
57		cis-calamenene	0.207	2008	34.944
58		δ -cadinene	2.663	2025	35.284
59		Cadina-1, 4-diene	0.103	2041	35.514
60		α –calacorene	0.087	2058	35.626
61		α –cadinene	0.163	2074	35.727
62		Ledane	0.092	2639	36.062
63		Epiglobulol	1.167	2668	36.509
64		β –maaliene	0.306	2698	36.612
65		Palustrol	0.190	2727	36.751
66		Spathlenol	1.915	2757	37.076
67	Globulol	5.997	2786	37.554	
68	Veridiflorol	1.243	2816	37.74	
69	1, 3-dimethyl-5-ethyladamantane	0.285	2845	37.80	
70	Ledol	0.753	2875	38.036	
71	γ- curcumene	0.391	2963	38.965	
72	Isospathulenol	0.300	2992	39.259	
73	Tau-muurolol	1.580	2509	39.495	
74	δ -cadinol	0.231	2529	39.562	
75	Guaia-3, 9-diene	0.292	2548	39.767	
76	α– cadinol	0.806	2568	39.908	
77	Vulgarol A	0.129	2587	40.375	
78	Hexadecanoic acid	0.093	2886	51.074	
79	2-tridecanol	0.028	2909	51.382	
80	Hexadecanoic acid ethyl ester	0.025	2932	51.755	
81	Decyltetraglycol	0.025	2955	59.356	
82	Tricosane	0.012	2979	61.218	
83	Benzonitrile, m-phenethyl	0.032	-	-	
84	Pentacosane	0.073	-	0.046	
85	Pentaethoxylated pentadecyl alcohol	0.036	-	-	
86	1-cyclohexene-1-carboxaldehyde, 4-(1-methylethyl)	0.170	-	-	
87	Cyclohexene, 3-methyl-6-(1-methylethyl)	0.108	-	-	
88	2- cyclohexene-1-ol, 2-methyl-5-(1-methylethenyl)-, trans-	0.059	-	-	
89	2, 3-dimethyl-cyclohexa-1, 3-diene	0.390	-	-	
90	α –campholonic acid	0.049	-	-	
91	Furan, 2, 3-dihydro-4-(1-methylpropyl)	0.458	-	-	
92	(E)-3-isopropyl-6-oxo-2-heptenal	0.058	-	-	
93	1, 5, 5-trimethyl-6-methylene- cyclohexene	0.056	-	-	
94	2, 6, 10-trimethyl-2, 5:7, 10-dioxido- dodeca-3, 11-diene-5-ol	0.268	-	-	
95	Tricyclo [6.3.0.1(2, 3)] undec-7-ene, 6, 10, 11, 11-tetramethyl	0.138	-	-	
96	1-methyl-4-isopropyl-cis-3- hydroxycyclohex-1-ene-6-one	0.230	-	-	
97	1H-cyclopropa[a]naphthalene, decahydro- 1,1,3 a-trimethyl-7-methylene-, [1as(1a.1alpha.,3a.alpha.,7a.beta.,7b.alpha.)]	0.235	-	-	
98	Naphthalene, 1, 2, 3, 4, 4a, 7- hexahydro-1, 6- dimethyl-4-(1-methylethyl)	0.139	-	-	
99	Bicyclo[3.1.0]hex-2-ene,2-methyl-5- (1-methylethyl)	0.026	-	-	
100	(+)-(1R, 2S, 4R, 7R)-7-isopropyl-5- methyl-5- bicycle [2.2.2] octen-2-ol	0.140	-	-	
101	1, 6-dimethyl-2-cyano-3-ethyl-3- piperidine	0.612	-	-	

1 Compound percentage

2 Retention index

3 Retention time

**Table 2:** Composition of the volatile oil of *Eucalyptus microtheca *flowers

**No**	**Compound**	**%** 1	**RI** 2		**RT** 3 ** (min)**
1	α –thujene	0.504	817		9.331
2	α -pinene	16.246	841		9.652
3	α -fenchene	0.078	866		9.976
4	Comphene	0.271	891		10.028
5	Verbenene	0.051	916		10.198
6	β - pinene	11.082	940		11.256
7	β -myrcene	0.263	955		11.957
8	α -phellandrene	7.006	976		12.477
9	α -terpinene	0.367	997		12.983
10	o- cymene	13.522	1018		13.246
11	Sabinene	2.131	1038		13.465
12	Limonene	2.713	1059		13.586
13	*cis-*ocimene	0.149	1080		13.993
14	γ -terpinene	0.868	1101		14.857
15	Isopropenyltoluene-cymene	0.093	1122		15.942
16	α -terpinolene	0.189	1143		16.195
17	Linalool L	0.058	1151		16.669
18	Appel oil	0.113	1170		16.956
19	D-fenchyl alcohol	0.085	1190		17.108
20	Hexadecane	0.147	2639		38.511
21	*Trans*-pinocarveol	0.365	1229		18.155
22	Pinocarvone	0.303	1248		18.779
23	4-methyl-1,3-heptadiene (c,t)	0.088	1267		19.161
24	2, 4-hexadiene, 2, 5-dimethyl-	0.070	1287		19.351
25	4-terpineol	1.052	1306		20.011
26	Myrtenal	0.202	1326		20.218
27	α -terpineol	0.425	1345		20.561
28	Myrtenol	0.160	1365		20.916
29	Dodecane	0.392	1408		21.584
30	β -citronellol	0.365	1428		22.624
31	Piperitone	0.167	1448		22.879
32	Citrol	0.063	1487		23.727
33	Citronellyl formate	0.115	1507		24.60
34	Diglycol dimethacrylate	0.787	1527		25.673
35	Carvacrol	0.494	1546		25.898
36	2-butylpyridine	0.129	1750		29.074
37	Isoledene	0.170	1779		29.249
38	Copaene	0.150	1809		29.322
39	Tetradecane	0.063	1839		29.463
40	β -elemene	0.063	1615		29.933
41	α -gurjunene	0.542	1631		30. 707
42	Seychelene	0.040	1647		30.833
43	*Trans*-Caryophyllene	0.227	1664		30.967
44	γ -selinene	0.122	1680		31.272
45	Calarene	0.112	1697		31.524
46	β - gurjunene	0.073	1713		31.621
47	Aromadendrene	7.444	1729		31.901
48	α -humulene	0.080	1746		32.31
49	Alloarmadendrene	1.632	1762		32.619
50	α -amorphene	0.400	1779		33.272
51	β –selinene	0.311	1795		33.58
52	α -guaiene	0.320	1811		33.744
53	Ledene	2.135	1828		34.051
54	α –muurolene	0.318	1844		34.225
55	γ -cadinene	0.667	1861		34.686
56	Calamenene	0.248	1877		34.81
57	δ -cadinene	1.040	1893		l
58	Cadina-1, 4-diene	0.045	1910		35.395
59	α -calacorene	0.070	1926		35.507
60	Epiglobulol	0.975	2374		36.334
61	β -maaliene	0.253	2403		36.482
62	Plustrol	0.221	2433		36.637
63	Spathlenol	1.848	2462		36.864
64	Globulol	5.419	2492		37.288
65	Veridiflorol	1.044	2521		37.497
66	Ledol	0.631	2580		37.867
67	Hexadecane	0.212	2698		38.735
68	α -ylangene	0.196	2727		38.826
69	Isospathulenol	0.217	2757		39.071
70	Tau-cadinol	0.791	2786		39.268
71	α -cadinol	0.444	2372		39.708
72	Cadalene	0.120	2392		40.257
73	N-octadecane	0.246	3211		45.874
74	Tetradecanamide	0.321	2653		50.157
75	n-hexadecanoic acid	0.375	2676		50.804
76	Ecosane	0.167	2700		52.389
77	Hexaadecanamide	0.918	2723		56.50
78	Octadecanoic acid	0.425	2746		56.848
79	Docosan	0.145	2769		58.363
80	9-octadecenamide	5.414	2793		61.623
81	Di-[2-ethylhexyl] phthalate	0.584		2816	66.492
82	4-methylenespiro[2,4]heptane	0.055		1209	17.288
83	(2-methylprop-1-enyl)-cyclohexa- 1, 3-diene	0.098		1467	23.271
84	1-(2′-hydroxy-3′,4′-dimethylphenyl) ethanone	0.603		2668	38.611
85	Trans-1,6-dimethyl bicycle (4.3.0) non-2-en-7-one	0.346		2551	37.595
86	7, 9-di-tert-butyl-1-oxaspiro [4.5] deca-6, 9- diene-2, 8-dione	0.117		2630	48.094
87	1, 3- cyclohexadiene, 2-methyl-5-(1-methylethyl), monoepoxide	0.139		1384	21.004
88	1H-cyclopropa[e]azulene, decahydro-1, 1, 7-trimethyl-4-methylene-,[1aR (1a.1alpha. 4a.beta. 7b.alpha)] - 7.alpha, 7a.beta	0.243		2610	38.049

1 Compound percentage

2 Retention index

3 Retention time

The presence of monoterpenes (60.899%), sesquiterpenes (28.328%), polyketides and fatty acids (1.714%), alkanes (1.372%), amides (6.653%), aromatic (0.115%), and other compounds (0.871%) was revealed for *E. microtheca* flower oils. In *E. viminalis* leaf oils, monoterpenes (83.037%) were the major components followed by sesquiterpenes (14.97%) and other minor components such as polyketides and fatty acids (0.496%), alkanes (0.046%), aromatic compounds (0.013%), and other compounds (1.404%). 

The results showed in the essential oil of* E. microtheca* leaves, 101 compounds representing 100%, were identified. Among them, α-phellandrene (16.487%), aromadendrene (12.773%), α-pinene (6.752%), globulol (5.997%), ledene (5.665%), P-cymen (5.251%), and β-pinene (5.006%) were the major constituents (Table 1).

In the oil of* E. microtheca* flowers, 88 compounds representing 100%, were identified in which α-pinene (16.246%), O-cymen (13.522%), β-pinene (11.082%), aromadendrene (7.444%), α-phellandrene (7.006%), globulol (5.419%), and 9-octadecenamide (5.414%) were the major components (Table 2). Sixty six compounds representing 100% were identified in the essential oil of* E. viminalis* leaves. The major compounds were 1, 8-cineole (57.757%), α-pinene (13.379%), limonene (5.443%), and globulol (3.054%) (Table 3).

**Table 3:** Composition of the volatile oil of *Eucalyptus viminalis *leaves

**No**	**Compound**	**%** 1	**RI** 2	**RT** 3 ** (min)**
1	Pinocarvone	0.085	1345	18.832
2	2, 5-octadiene	0.068	1365	19.353
3	δ -terpineol	0.070	1384	19.461
4	Borneol	0.076	1404	19.593
5555	4-terpineol	0.722	1423	20.031
6	P-cym-8-ol	0.039	1443	20.269
7	β -fenchyl alcohol	0.983	1462	20.703
8	P-mentha-1, 8-dien-3-one	0.071	1487	21.97
9	5, 6-decanedione	0.036	1720	26.596
10	Copaene	0.059	1750	29.359
11	Methyleugenol	0.025	1779	29.59
12	Eudesma-3, 7(11)-diene	0.038	1582	30.196
13	α -gurjunene	1.372	1598	30.779
14	Valencene	0.094	1615	31.309
15	Calarene	0.407	1631	31.567
16	Selina-3, 7 (11)-diene	0.057	1647	31.657
17	Aromadendrene	3.925	1664	31.949
18	Alloarmadendrene	2.023	1680	32.707
19	Isoamyl phenyl acetate	0.202	1697	33.12
20	β -selinene	0.156	1713	33.617
21	Ledene	0.639	1729	34.089
22	α - muurolene	0.089	1746	34.258
23	γ -cadinene	0.201	1762	34.723
24	calamenene	0.279	1779	34.848
25	δ -cadinene	0.233	1795	35.119
26	Epiglobulol	0.555	2138	36.403
27	γ –gurjunene	0.169	2168	36.538
28	Palustrol	0.142	2197	36.688
29	Globulol	3.054	2227	37.369
30	Veridiflorol	0.881	2256	37.586
31	1, 3-dimethyl-5-ethyladamantane	0.250	2286	37.674
32	*Trans*- β -farnesene	0.070	2374	38.289
33	α –cadinol	0.106	2138	39.765
34	Citronellyl acetate	0.063	2158	42.108
35	N-hexadecanoic acid	0.030	2327	50.917
36	Pentacosane	0.046	2351	66.562
37	Octanal	0.019	866	7.611
38	2-methyl-1, 3-cycloheptadiene	0.041	891	8.907
39	α -thujene	0.035	916	9.373
40	α -pinene	13.379	940	9.732
41	α -fenchene	0.018	965	10.009
42	comphene	0.063	990	10.055
43	β - pinene	0.555	1014	11.191
44	β -myrcene	0.857	1018	12.001
45	α -phellandrene	0.169	1038	12.443
46	o- cymene	0.118	1059	13.283
47	1, 8-cineole	57.757	1080	13.919
48	Limonene	5.443	1101	13.98
49	Cis-ocimene	0.013	1122	14.113
50	β - ocimene Y	0.011	1143	14.56
51	isoamyl butyrate	0.013	1164	14.686
52	γ –terpinene	0.514	1185	14.941
53	Dehydro-p-cymen	0.094	1206	16.012
54	α -terpinolene	0.771	1209	16.276
55	Linalool L	0.099	1229	16.784
56	Appel oil	0.668	1248	17.031
57	Isoamyl valerate	0.028	1267	17.14
58	Fenchol	0.035	1287	17.275
59	Valeric acid 4-pentenyl ester	0.119	1306	17.407
60	*Trans*-pinocarveol	0.212	1326	18.248
61	(+)-(2S, 4R)-p-mentha- 1(7), 8-dien-2-ol	0.067	1507	22.283
62	1H-indene, 1-ethylideneoctahydro-7a-methyl-, (1E, 3a.alpha. 7a.beta)	0.466	2315	37.931
63	Bicyclo [4.4.0] dec-1-ene, 2- isopropyl-5-methyl-9-methylene	0.191	2433	39.327
64	Caryophylla-2(12), 6(13)-dien-5-one	0.230	2344	38.107
65	1-(2′-hydroxy-3′,4′-dimethylphenyl) ethanone	0.598	2403	38.692
66	2-propenoic acid, 2-methyl-,1,2-ethanediyl ester	0.068	1527	25.832

1 Compound percentage

2 Retention index

3 Retention time

**Table 4:** Comparison of the composition of the volatile oil of *E. microtheca *leaves and flowers with *E. viminalis *leaves from Zahedan

**No**	**Compound**		**%** 1		**%** 2		**%** 3	
1	α –thujene		0.742		0.504		0.035	
2	α -pinene		6.752		16.246		13.379	
3	Comphene		0.079		0.271		0.63	
4	β - pinene		5.006		11.082		0.555	
5	β -myrcene		0.533		0.263		0.857	
6	α -phellandrene		16.487		7.006		0.169	
7	α -terpinene		0.832		0.367		-	
8	P- cymene		5.251		-		-	
9	β -phellandrene		2.194		-		-	
10	Limonene		1.503		2.713		5.443	
11	Cis-ocimene		1.655		0.149		0.013	
12	β –ocimene Y		0.101		-		0.011	
13	γ -terpinene		1.235		0.868		0.514	
14	Cymene		0.024		-		-	
15	α -terpinolene		0.425		0.189		0.771	
16	Rosefuran		0.024		-		-	
17	Cycloheptanmethanol		0.061		-		-	
18	Linalool L		0.093		0.058		0.099	
19	Isoamyl isovalerate		0.529		-		-	
20	Isoamyl valerate		0.056		-		0.028	
21	Fenchol		0.076		-		-	
22	*Trans*-pinene hydrate		0.062		-		-	
23	Allocimene		0.049		-		-	
24	1-terpineol		0.045		-		-	
25	1-methylnorcarane		0.051		-		-	
26	Ethylbenzoate		0.124		-		-	
27	4-terpineol		1.256		1.052		0.722	
28	1-(adamantly) cyclohexene		0.042		-		-	
29	β -fenchol		0.203		-		-	
30	*Cis*-sabinol		0.224		-		-	
31	Thiophene, 2-ethyl-5-methyl		0.120		-		-	
32	Ascaridole		0.085		-		-	
33	Dicyclobutylidene oxide		0.084		-		-	
34	Divinyldimethylsilane		0.114		-		-	
35	Piperitone		0.196		-		-	
36	1-methoxyhept-1-yne		1.809		-		-	
37	Citronellyl formate		0.029		0.115		-	
38	Carvacrol		0.420		0.494		-	
39	α** –**cubebene		0.160		-		-	
40	Isoledene		0.278		0.170		-	
41	Copaene		0.308		0.150		0.059	
42	2-pentene-1-ol, 2-methyl		0.215		-		-	
43	α –gurjunene		1.897		0.542		1.372	
44	*Trans*-caryophyllene		0.539		0.227		-	
45	Aromadendrene		12.773		7.444		3.925	
46	Epizonaren		0.067		-		-	
47	α –humulene		0.142		0.080		-	
48	alloarmadendrene		2.520		1.632		2.023	
49	γ –gurjunene		0.327		-		0.169	
50	α –copaene		0.755		-		-	
51	β –selinene		0.525		-		0.156	
52	β –panasinsene		0.702		-		-	
53	ledene		5.665		-		0.639	
54	α –muurolene		0.398		-		0.089	
55	Geremacrene B		0.099		-		-	
56	α –amorphene		1.666		0.4		-	
57	*cis*-calamenene		0.207		-		-	
58	δ -cadinene		2.663		-		0.233	
59	Cadina-1, 4-diene		0.103		0.045		-	
60	α –calacorene		0.087		0.070		-	
61	α –cadinene		0.163		-		-	
62	Ledane		0.092		-		-	
63	Epiglobulol		1.167		0.975		0.555	
64	β –maaliene		0.306		0.253		-	
65	Palustrol		0.190		0.221		0.142	
66	Spathlenol		1.915		1.848		-	
67	Globulol		5.997		5.419		3.054	
68	Veridiflorol		1.243		1.044		0.881	
69	1, 3-dimethyl-5-ethyladamantane		0.285		-		0.250	
70	Ledol		0.753		0.631		-	
71	γ- curcumene		0.391		-		-	
72	Isospathulenol		0.300		0.217		-	
73	Tau-muurolol		1.580		-		-	
74	δ -cadinol		0.231		-		-	
75	Guaia-3, 9-diene		0.292		-		-	
76	α– cadinol		0.806		0.444		0.106	
77	Vulgarol A		0.129		-		-	
78	Hexadecanoic acid		0.093		0.375		0.030	
79	2-tridecanol		0.028		-		-	
80	Hexadecanoic acid ethyl ester		0.025		-		-	
81	Decyltetraglycol		0.025		-		-	
82	Tricosane		0.012		-		-	
83		Benzonitrile, m-phenethyl		0.032		-		-	
84		Pentacosane		0.073		-		0.046	
85		Pentaethoxylated pentadecyl alcohol		0.036		-		-	
86		1-cyclohexene-1-carboxaldehyde, 4-(1-methylethyl)		0.170		-		-	
87		Cyclohexene, 3-methyl-6-(1-methylethyl)		0.108		-		-	
88		2- cyclohexene-1-ol, 2-methyl-5-(1-methylethenyl)-, trans-		0.059		-		-	
89		2, 3-dimethyl-cyclohexa-1, 3-diene		0.390		-		-	
90		α –campholonic acid		0.049		-		-	
91		Furan, 2, 3-dihydro-4-(1-methylpropyl)		0.458		-		-	
92		(E)-3-isopropyl-6-oxo-2-heptenal		0.058		-		-	
93		1, 5, 5-trimethyl-6-methylene- cyclohexene		0.056		-		-	
94		2, 6, 10-trimethyl-2, 5:7, 10-dioxido- dodeca-3, 11-diene-5-ol		0.268		-		-	
95		Tricyclo [6.3.0.1(2, 3)] undec-7-ene, 6, 10, 11, 11-tetramethyl		0.138		-		-	
96		1-methyl-4-isopropyl-cis-3- hydroxycyclohex-1-ene-6-one		0.230		-		-	
97		1H-cyclopropa[a]naphthalene, decahydro- 1,1,3a-trimethyl-7-methylene-, [1as(1a.1alpha.,3a.alpha.,7a.beta.,7b.alpha.)]		0.235		-		-	
98		Naphthalene, 1, 2, 3, 4, 4a, 7- hexahydro-1, 6- dimethyl-4-(1-methylethyl)		0.139		-		-	
99		Bicyclo[3.1.0]hex-2-ene,2-methyl-5- (1-methylethyl)		0.026		-		-	
100		(+)-(1R, 2S, 4R, 7R)-7-isopropyl-5- methyl-5- bicycle [2.2.2] octen-2-ol		0.140		-		-	
101		1, 6-dimethyl-2-cyano-3-ethyl-3- piperidine		0.612		-		-	

1 *E. microtheca *leaves

2 *E. microtheca *flower

3 *E. viminalis *leave

## Discussion

The comparison of results showed that there are some differences and similarities between the oil compositions of these *Eucalyptus *species. These results are shown in Table 4. The percentages of sesquiterpene and monoterpene compounds were similar in *E. microtheca* leave oils, but the percentages of these components were less than those of* E. viminalis* leave and *E. microtheca *flower oil. Studies have revealed that monoterpenes have insecticidal activities against the stored–product insects (Rajendran and Sriranjini, 2008 ▶; Papachristos et al., 2004 ▶). Our study showed that the major monoterpene compounds were in *E. viminalis* leave and *E. microtheca *flower oil. These compounds consist of 1, 8- cineole, α- pinene, and β-pinene which have been shown to have insecticidal effects against some major insects that infect the stored crops (Rajendran and Sriranjini, 2008 ▶). Therefore, the essential oil of *E. viminalis* leaves and *E. microtheca *flowers from Zahedan, Iran could be a valuable alternative to chemical control strategies which have undesirable effects such as environmental pollution and direct toxicity to people. As it is evident from Table 3, the main component of the essential oils of *E. viminalis* leaves was 1, 8-cineole (57.757%), but it was not identified in *E. microtheca* leaf and flower oils*.* 1, 8-cineole, which is a terpenoid oxide present in many plant essential oils, displays anti-microbial, anti-inflammatory, and anti-nociceptive effects (Juergens et al., 2003 ▶; Santos and Rao, 2000 ▶).

The percentage of α-pinene in the oil of *E. microtheca *flowers and* E. viminalis* leaves was 16.246% and 13.379%, respectively, while in *E. microtheca* leave oil it was less than 10%. Results indicated that some of* E. microtheca* leaf oil compounds such as α-phellandrene (16.487%) and aromadendrene (12.773%) were higher compared with* E. microtheca *flower and *E. viminalis* leave oils. The oil of *E. microtheca* flower contained β-pinene (11.082%), while it was less than 10% in other oils (*E. microtheca* and *E. viminalis* leave oil). The compounds such as α-pinene and β-pinene were the main components in the essential oil of* E. microtheca* flowers (16.246% and 11.082%) and *E. viminalis* leaves (13.379% and 0.555%), respectively. These compounds have been proven to be strong antioxidant and antimicrobial agents as emphasized elsewhere (Ho, 2010 ▶).

Chemical composition of the essential oil of *Eucalyptus microtheca* leaves growing in different geographical locations has been widely studied. Ogunwande et al., (2003) ▶ reported that in the volatile oil of *Eucalyptus microtheca* leaves from Nigeria, 1, 8-cineole (53.80%) was the main constituent in leaves (Ogunwande et al., 2003 ▶). Sefidkon et al., (2007) ▶ identified 22 components in the oil of *E. microtheca* from Kashan in the central region of Iran. The major components were 1, 8-cineole (34.0%), P-cymene (12.40%), α-pinene (10.70%), β-pinene (10.50%), and virdiflorene (5.20%) (Sefidkon et al., 2007 ▶). In another study, the major constituent of *E. microtheca* leaf oils from Semnan province was 1, 8-cineole (48.51%), followed by aromadendrene (18.31%), α-pinene (9.47%), and alloaromadendrene (4.67%) as the other dominant constituent (Hashemi-Moghaddam et al., 2013 ▶). There are many references about the composition of other *Eucalyptus *species in the literature. For example, the main constituents of the oil of *E. sargentii* from Isfahan province were 1, 8-cineole (55.48 %), α-pinene (20.95 %), aromadendrene (6.45 %), and *trans*-pinocarveol (5.92%) (Safaei and Batooli, 2010 ▶). Assareh et al., (2007) ▶ also reported chemical composition of the essential oils of six *Eucalyptus* species from South West of Iran. The main components identified in *E. intertexta* oil were 1, 8-cineole (64.80%), terpinen-1-ol (7.20%), and α-pinene (5.70%); in *E. largiflorens* were 1, 8-cineole (47.0%), P-cymene (10.60%), and α-terpineol (8.50%); in *E. kingsmillii* were 1, 8-cineole (77.0%), α-pinene (8.70%), and camphene (3.80%); in *E. dealbata* were 1, 8-cineole (70.60%), α-pinene (13.0%), and terpinen-1-ol (3.70%). The major components of the oil of *E. loxophleba* were 1, 8-cineole (41.90%), α-pinene (13.70%), and aromadendrene (3.70%), while the major components of *E. kruseana* were bicyclogermacrene (28.80%), α-pinene (17.70%), and 1, 8-cineole (12.10%) (Assareh et al., 2007 ▶). Abd El- Mageed et al., (2011) ▶ identified chemical composition of the essential oils of some *Eucalyptus* species from Egypt. The major components identified in *E. citridora* oil were 3-hexen-1-ol (31.26%), *cis*-geraniol (19.66%), citronellol acetate (13.68%), 5-hepten-1-ol, 2, 6-dimethyl (13.14%), and citronellal (9.36%); in *E. gomphocephala* were dihydrocarveol acetate (50.82%) and P-cymene (10.62%); and the major components of *E. resinfera* were eucalyptol (51.97%), spathulenol (9.22%), α-terpineol acetate (8.78%), and *trans*-nerolidol (8.75%) (Abd El- Mageed et al., 2011 ▶). Mubarak et al., (2014) ▶ reported γ-terpinene (71.36%) and O-cymene (17.63%) as the major components of *E. camaldulensis *from Malaysia (Mubarak et al., 2014 ▶)*.* Comparing the results of different studies showed that although 1, 8-cineole has not been identified in *E. microtheca* leaf and flower oil from Zahedan, but it was as the major constituent of *E. microtheca* leaf oil from Nigeria (53.80%), Semnan (48.51%), Kashan (34.0%), and other* Eucalyptus *species (*E. kingsmillii* 77.0%, *E. dealbata* 70.60%, *E. intertexta* 64.80%, *E. viminalis* 57.75%, *E. sargentii* 55.48%, *E. largiflorens* 47.0%, and *E. loxophleba* 41.90%). The essential oil of some *Eucalyptus *species rich in 1, 8-cineol are widely used as a flavoring agent in production of softeners, soap, toothpaste, and other medicines (Sefidkon et al., 2007 ▶), but the percentage of this compound is different in species. This can be related to the type of the plant, the plant parts (aerial or flower and leaf parts), the geographical regions of the plant growing places, and also the ecological conditions of the plant. In addition, α-pinene compound, which appeared as the major constituent in the oil of *E. sargentii* (20.95%), *E. kruseana* (17.70%),* E. viminalis *(13.379%),* E. loxophleba *(13.70%), *E. dealbata* (13.0%), and *E. microtheca* from Kashan (10.70%) and Semnan (9.47%), were present in low concentration in* E. microtheca* leaf oils (6.752%) from Zahedan. The amount of P-cymene compound in the oil of *E. microtheca* leave from Kashan (12.40%) also was much higher than that of *E. gomphocephala* (10.62%), *E. largiflorens* (10.60%), and *E. microtheca* (5.21%) from Zahedan. 

In general, great quantitative and qualitative variations in volatile composition of *E. viminalis* and *E. microtheca* were seen between this and other studies. These variations may be due to the influence of geographical differences, environmental and growing conditions, physiological and biochemical states of plants, genetic factors, and different extraction and analytical procedures (Kokkini et al., 2004 ▶; Hassanpouraghdam et al., 2011 ▶). 

It can be concluded that the oils of these two *Eucalyptus *species are good sources of natural antioxidants to be used in medicinal and food products to promote human health and prevent diseases, which should be investigated in further studies. In addition, regarding environmental problem and human health, these plants could be an alternative source of insecticide agents because many of their components have little or no harmful effects on humans and environment.
